# Development of a practical guideline for person centred goal setting in geriatric rehabilitation: a participatory action research

**DOI:** 10.1007/s41999-023-00830-w

**Published:** 2023-07-17

**Authors:** Elizabeth M. Wattel, Aafke J. de Groot, Sacha Deetman-van der Breggen, Robin Bonthuis, Niels Jongejan, Marina M. R. Tol-Schilder, Johannes C. van der Wouden, Robbert Gobbens

**Affiliations:** 1 Department of Medicine for Older People, Amsterdam UMC, Location Vrije Universiteit Amsterdam, De Boelelaan 1109, OZW 8B-05, 1081 HV Amsterdam, The Netherlands; 2 Aging and Later Life, Amsterdam Public Health, Amsterdam, The Netherlands; 3 Vivium Zorggroep, Naarden, The Netherlands; 4 Zonnehuisgroep IJssel-Vecht, Location Stadshagen, Geriatric Rehabilitation, Zwolle, The Netherlands; 5 Stichting QuaRijn, Geriatric Rehabilitation Care, Doorn, The Netherlands; 6 Cordaan, Amsterdam, The Netherlands; 7 Zonnehuisgroep Amstelland, Amstelveen, The Netherlands; 8 Faculty of Health, Sports and Social Work, Inholland University of Applied Sciences, Amsterdam, The Netherlands; 9 Department Family Medicine and Population Health, Faculty of Medicine and Health Sciences, University of Antwerp, Antwerp, Belgium; 10 Tranzo, Tilburg University, Tilburg, The Netherlands

**Keywords:** Geriatric rehabilitation, Patient centeredness, Goal setting, Practical Guideline

## Abstract

**Purpose:**

To improve goal setting in Geriatric Rehabilitation (GR), by developing an evidence-based practical guideline for patient-centred goal setting.

**Methods:**

Participatory action research (PAR) in a cyclical process, with GR professionals as co-researchers. Each cycle consisted of five phases: problem analysis, literature review, development, practical experience, feedback & evaluation. The evaluation was based on video recordings of goal setting conversations, and on oral and written feedback of the GR professionals who tested the guideline.

**Results:**

In two PAR-cycles the guideline was developed, consisting of eight recommendations for setting and using goals, and of practical advices elaborating three of the recommendations, concerning conversational skills specific for goal setting conversations. After the second cycle the research team concluded that the guideline was feasible in daily practice and effective when used consciously.

**Conclusion:**

In this study, a practical guideline for setting and using goals in GR was developed. GR teams can improve their patient centred working with goals by discussing the recommendations in their team and choosing the recommendations to work on. This can be supported by the development of an interdisciplinary training. The effect on quality of care should be subject to further investigation.

**Supplementary Information:**

The online version contains supplementary material available at 10.1007/s41999-023-00830-w.

## Key summary points

**Aim:**

Developing an evidence based practical guideline for patient-centred goal setting in geriatric rehabilitation.

**Findings:**

The guideline consists of eight recommendations, including three recommendations concerning conversational skills. Those three are further elaborated into practical advices.

**Message:**

Geriatric rehabilitation teams can improve their patient centred working with goals by discussing the recommendations in their team and choosing the recommendations to work on.

**Supplementary Information:**

The online version contains supplementary material available at 10.1007/s41999-023-00830-w.

## Introduction

Older people with a (sub)acute deterioration in functioning, caused by for example stroke or hip fracture, can benefit from geriatric rehabilitation (GR) [1]. GR is a multidimensional approach consisting of diagnostic and therapeutic interventions, the purpose of which is to optimize functioning and participation [2, 3]. GR starts off with a comprehensive geriatric assessment, that aims to identify the specific problems and needs of a patient. Subsequently, rehabilitation goals are drawn up, which form the basis of the patient specific multidisciplinary rehabilitation plan [3]. In the Netherlands, every year about 45,000 older persons are admitted to GR after hospitalization [4].

Ideally the rehabilitation goals are set in dialogue with the patient. This process of establishing or negotiating rehabilitation goals is called ‘goal setting’ [5]. Research in adult rehabilitation patients, who suffer less from geriatric syndromes and comorbidity, shows low quality evidence that goal setting leads to better psychosocial outcomes [5]. These findings are not yet confirmed in GR literature, where the research mainly focusses on effects on length of stay and functioning at GR discharge [6]. Possibly goal setting in GR merely improves other outcomes such as patient satisfaction and self-efficacy [7]. Goal setting fits in the modern standards of good care that concerns the whole person, including preferences, experiences, and the right to make decisions about one’s own treatment [8]. This is consistent with the fact that person centred care and goal setting are key elements in Dutch governmental documents on good care and the Dutch research agenda for GR [9, 10].

Although both professionals and patients think it is important to genuinely involve GR patients and their informal caregivers in goal setting, in daily practice it appears to be difficult [11–13]. Patients feel that goals are mainly set by professionals and rehabilitation professionals have doubts about the capability of GR patients to formulate realistic goals, although even in patients with mild to moderate dementia collaborative goal setting appears to be feasible [11–14]. Complicating factors are the fact that not all patients aspire the same active role in the goal setting process and the desired role can change over time [15]. Another aspect that hinders the enhancement of goal setting is that goal setting is generally new to patients in GR and that they have difficulties in understanding what is expected from them [16]. Finally, GR professionals tend to overestimate the patient’s influence in their own goal setting, and at the same time, many patients rate their involvement in the establishment of their goals as insufficient [11, 16, 17].

Within the University Network of Care for Older People Amsterdam (UNO Amsterdam), researchers of Amsterdam UMC and health care professionals collaborate to connect research and daily practice. In the GR working group of UNO Amsterdam, GR professionals of the network (e.g., physicians, nurses and therapists) and researchers have chosen goal setting as a topic where research can help to improve their daily GR practice, and thereby improve the quality of care.

To improve goal setting in GR, we aimed to develop a practical guideline for patient-centred goal setting. To target the problems that GR professionals experience in the performance of goal setting, this study was conducted in co-creation with GR professionals connected to UNO Amsterdam.

## Methods

### Design

We chose a participatory action research (PAR) design [18, 19], in which a practical guideline was developed, tested and evaluated in collaboration with professionals involved in GR. We reported according to the recommendations of Smith et al. [20]

### Participants

UNO is a network in which 23 care organizations throughout the Netherlands collaborate to connect scientific research and daily practice in the field of care for older adults with vulnerability. Members of the GR commission of UNO Amsterdam (both GR professionals and researchers) initiated this study and participated in it as the PAR research team. The testing of the guideline was performed by the GR professionals (e.g., physicians, nurses and therapists) of the research team, and / or by their colleagues in their GR organization.

### Participatory action research (PAR)

PAR is an approach that is suited to improve practice in co-creation between researchers and professionals. [18] We used the definition of Van Buul et al.: “Participatory action research aims to bring about change in social situations by both *improving practice* (i.e. taking action) and *creating knowledge or theory* (i.e. reflecting on action).”[…] “It works through a *cyclical process* of planning, action and reflection. This process is *collaborative*: it requires substantial involvement of relevant stakeholders, which facilitates empowerment. The persons under study are considered ‘co-researchers’ who test practices and gather evidence in action phases, and evaluate this action and plan further action in reflection phases.” [19].

**Fig. 1:**
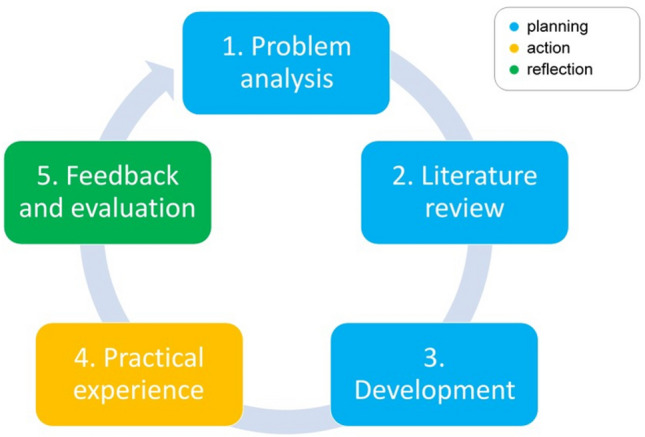
Cyclical process of PAR

In the current study, the cyclical process started with addressing the challenges of goal setting in GR as an important problem. Each cycle consists of five phases, each representing an element of the cyclical process of planning, action and reflection that is typical of PAR (Fig. 1). Depending on the evaluation in the fifth phase a new cycle started or the cyclical process was terminated.

An extensive description of the PAR-phases is presented in Online Resource A. In summary the phases include:

#### Phase 1: problem analysis 

Members of the research team analyzed the goal setting process combining both practical experience and literature research, and formulated the problems that had to be solved.

#### Phase 2: literature review

Members of the research team searched scientific literature to solve the problems found in phase 1.

#### Phase 3: development

Members of the research team discussed the scientific literature to develop and subsequently adapt the guideline for goal setting in GR.

#### Phase 4: practical experience

GR professionals (members of the research team and their colleagues in the GR organization) tested the guideline in their daily practice and provided feedback.

#### Phase 5: feedback and evaluation

Members of the research team reviewed the feedback and judged if the guideline was ready for dissemination and implementation in GR without further adjustments.

### Ethics approval

The study procedures were reviewed and approved by the Medical Ethics Review Committee of the VU University Medical Center (number 2020.492, Amsterdam, The Netherlands). The study was performed in line with the principles of the Declaration of Helsinki. [21]

### Consent

Written informed consent was obtained from all individual participants included in the study.

## Results

The guideline was created during the course of two cycles. In the first cycle, seven goal setting recommendations were developed and tested from February 2017 to February 2018. In the second cycle, practical advices for implementing three of the recommendations were developed from November 2019 to February 2022.

### Participants and their roles

The research team consisted of members of the GR commission of the UNO network: two elderly care physicians, a nurse practitioner, a nurse / professor, an occupational therapist/policy officer, a physiotherapist, and a researcher. Two elderly care physicians temporarily joined the team but had to quit due to time constraints. All members had extensive experience in GR and/or GR research (five to thirty five years). Five out of these nine participants were female. The members of the research team initiated the project, defined the research question, actively participated in all PAR-phases and in the writing of the research paper. The team was advised by a second researcher.

Colleagues of the research team members participated in the phase of practical experience, by testing the guideline and providing feedback. All were GR professionals. In the first cycle the professional background of the colleagues was not registered, in the second cycle they were three elderly care physicians, a resident, a nurse, a speech therapist, and three physiotherapists. Seven of them were female.

### Cycle 1: recommendations for goal setting

The problem analysis revealed that the rehabilitation plans are not adequately ‘owned’ by the patients and their relatives, and that this is due to a lack of tailored information, a lack of patient involvement in goal setting decision making and a lack of follow-up of the goals throughout the rehabilitation process, by the involved disciplines, the patients and their informal caregivers. This broadened the scope of the project to both goal setting and a visible role of the goals throughout the rehabilitation process. Based on the findings of Smit et al. [11] the research team concluded that developing recommendations, rather than a strict step-wise method, is preferred for enhancing goal setting practice. This allows GR teams to examine their performance on each recommendation, and only change their working method where improvement can be expected. The research team discussed Smit’s other findings, as well as studies on shared decision making within goal setting [16] and patient experiences of goal setting in post-acute stroke rehabilitation [15]. The discussion led to seven recommendations for goal setting in GR that were extended with an eighth recommendation in the second cycle. The recommendations are presented in Box 1.

Box 1. Recommendations for goal setting-dialogue in geriatric rehabilitation.
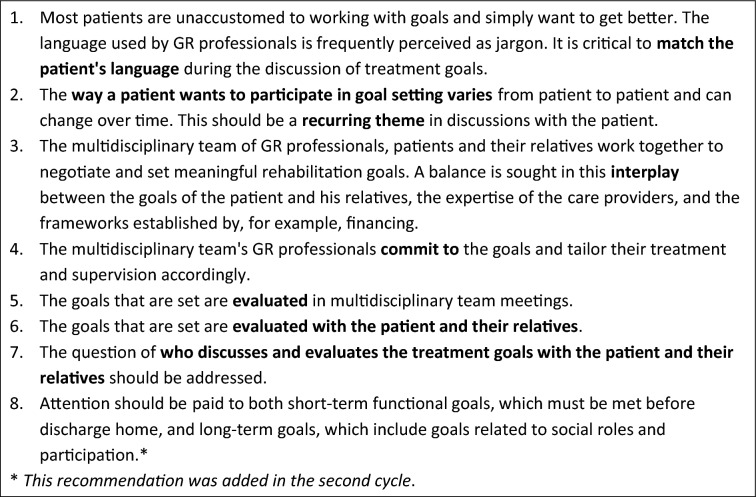


The testing of the recommendations revealed that, in general, the recommendations were deemed useful. The first three recommendations were thought to be more difficult to fulfil and needed practical elaboration. They concerned conversational skills that are specific for goal setting, for example, on the topic how to match the patient’s language and avoid using the word ‘goal’. The other four recommendations of the first cycle were thought to be more straightforward, because they only needed procedural adaptations. These recommendations concerned the key role of goals throughout the rehabilitation process. The first three recommendations were chosen to be addressed in the next cycle.

### Cycle 2: practical advices for implementing three of the recommendations

This cycle began by making video recordings of five real-life goal setting conversations with three female and two male patients: in all conversations a patient and a GR professional were present, the presence of other GR team members and relatives varied. In one conversation the goal setting process was structured by the Canadian Occupational Performance Measure (COPM) [22].

The scientific literature that was analyzed in this cycle concerned research on shared decision making in frail older adults [23–26], shared decision making within goal setting in rehabilitation [27], comprehensible communication [28], barriers and facilitators for goal setting [16], types of set goals [12, 13, 29–31] and the course of goal setting conversation and the interaction between patient and therapist [17, 30, 32–38]. Findings of these scientific publications were used to evaluate the video recordings. This resulted in the addition of an eighth recommendation to the set of recommendations and it resulted in practical advices for the goal setting conversation. The extra recommendation concerned the type of goals: not only functional goals that are needed for discharge, but also goals that focus on physical, functional and psychosocial aspects of life after discharge. The advices for the goal setting conversation were divided over three main elements: an introduction, the actual goal setting conversation and a summary and clarification of what has been discussed. The advices and the considerations that led to them are presented in Table 1.

**Table 1:** Practical advices for implementing recommendations 1, 2 and 3

Components of the goal setting conversation	Recommendation number, source and considerations
**Preparation questions for exploring the patient’s starting position:** • Have you previously received rehabilitation? (Are you familiar with the concept of goal setting?) • What did they tell you? • What do you require to recover?	**Recommendation**: 1,2 and 3 **Source**: video recordings, experience of research team and publications [7, 22, 24–26, 32, 36] **Considerations**: ‘Is this patient familiar with the concept of goal setting and emotionally ready for this conversation’ are important questions, entering this conversation. This is done by exploring expectations, knowledge, prior experience with rehabilitation and goal setting, and issues that distract from a goal setting conversation
**Explanation of the conversation’s purpose**: “This conversation is intended to cover two topics: 1. What do you need to be able to perform, to return home? 2. What else is necessary for you to be able to live at home again and to get your life back on track?”	**Recommendation**: 1 and 3 (matching the patient’s language & the interplay between participants leads to meaningful goals) **Source**: experience of research team and literature [11, 12, 15, 28, 36] **Considerations**: The distinction between functional goals that focus on discharge home and ‘other’ goals (e.g., participation goals, patient’s dreams, goals on cognition or mood) makes the goals more meaningful for the patients, and prevents from therapist-led choice of just ‘privileged goals’. [27]
**Explanation of the patient's role (patient is an expert on himself):** Briefly name options, e.g., “There are various ways to determine those rehabilitation goals. One way is for you to say what the goals are, another is for the doctors and therapists (that is “we”) to say what the goals are and a third way is for us to talk about it and decide together. Which do you prefer if I put it that way?”	**Recommendation**: 2 (opening the conversation about the patient’s desire to participate in decision making: how do you do that?) **Source**: video recordings and publications [22, 32, 36] **Considerations**: -
**Goal setting conversation, either COPM or other type**	**Recommendation**: 1, 2 and 3 **Source**: Publications [11, 12, 15, 17, 22, 24, 26, 28, 30, 31, 33–36] **Considerations**: The purpose of this cycle is not to choose the best goal setting intervention. For the Recommendation ‘the interplay between participants leads to meaningful goals’ interesting insights were found that can help the GR professionals improve the interplay between the goal setting participants - Use of a decision aid - For some patients it is helping to break down goals into smaller parts - Professionals prefer goals (‘privileged goals’) characterized by short timeframes, conservative estimation of outcomes, and physical function. The selection of other types of goals is unlikely - Patients goals deemed unattainable by the rehabilitation team are never agreed on - When professionals cannot agree on a patient's goals, they employ strategies such as: 1. Focusing on the admission rather than the long term if the possibility of success is uncertain; 2. Presenting information in a step-by-step manner to elicit agreement; 3. Indicating that the goal is essentially non-negotiable, for example, by writing it down, 4. Collaborating with other team members to formulate goals 5. Making use of the authority implicit in the professional role; 6. Moving on to the next goal despite signs of patient resistance - When patients use words like “Well….” or “I think ….” They might doubt if they are able to articulate goals
**Summary and clarification** • Summarize the goals and explain what disciplines are involved to reach the goals • Ask back if it is clear • Ask ‘What other questions do you have?’	**Recommendation**: 1. (matching the patient’s language) **Source**: video recordings, experience of research team and publications [17, 27] **Considerations**: The Pharos factsheet [26] emphasized the significance of these points in transferring the plan from the therapist’s head to the patient's. The final point ("What other questions do you have?") proved to be far more inviting than “Do you have any questions?”. Instead of being expected to understand everything, the patient is expected to have questions

Seven GR professionals put the practical advices to test and video recorded their goal setting conversations with five female and two male patients. In the assessment of the recordings it became clear that in three of them the GR professional obviously used the structure and practical advices. These conversations began with the proposed introduction, the use of the majority of the advices was identifiable and the professionals visibly checked the paper with the advices. The other four recordings did not show that the advices were used, the research team assumed this was, in retrospect, due to unclear instructions for the professionals. The research team decided to evaluate the feasibility and effect in the conversations where the advices were obviously used. These three professionals reported that the structuring effect of the advices supported them to have a successful goal setting conversation, despite the unnatural setting of the video recording. According to the GR professionals, two of the conversations resulted in clear rehabilitation goals for the patients. The third professional reported that the patient was unsure what to expect from her recovery and thus did not express her rehabilitation goals. This conversation was complicated by the fact that the GR professional was not the patient's actual therapist and thus could not contribute her professional perspective to the goal setting process. Based on the feedback of the GR professionals the research team concluded that the guideline (recommendations and practical advices) was feasible in daily practice and effective when used consciously.

### Next steps

The research team proposed to develop a training for the application of this guideline as a next step, and then perform a pilot study to test the effect of this training on patient involvement in goal setting and patient ownership of the rehabilitation process.

### The PAR process

Participatory action research in itself has a greater yield than just the results of the PAR-cycles. This is, for example, personal outcomes for the participants and learning points from challenges in the PAR process. The co-researchers experienced that their participation in this project raised their awareness of goal setting challenges. Their involvement made them aware of difficulties in choosing the right language, and of the fact that patients are not aware how the goals affect their rehabilitation trajectory.

An important challenge was the transparency and clarity of appointments for the research team members, due to the collective approach and the shared responsibility of the team members. An illustration of this was the preparation of the practical experience (phase 4) in the second cycle, where the advices for the goal setting conversation would be tested. As a team we thought that all team members knew what to do, but in the end, we had to conclude that four out of seven professionals that tested the goal setting conversation advices, got insufficient instructions.

What we learned from this challenge, is that appointments, goals and expectations have to be clear and unambiguous, and well-documented. And besides that, a clear training program is necessary for proper application of the practical guideline.

## Discussion

In this PAR-project, we aimed to develop a practical guideline for goal setting in GR. The resulting guideline consists of eight recommendations and a further practical elaboration of three of them, concerning conversational skills that are specific for goal setting. Multidisciplinary teams of GR professionals could use these recommendations for a tailored improvement of their patient centred goal setting practice.

An important choice in this study is the development of a practical guideline built on recommendations instead of an extensive step-by-step method for goal setting. This choice was based on the feasibility study of Smit et al. which showed that professionals failed to implement all parts of the planned intervention [11]. This finding is consistent with that of Scobbie et al. who studied the implementation of a step-by-step intervention for goal setting, and concluded that the new and unfamiliar steps were not routinely implemented [7]. This is supported by the key findings of Peryer et al. about implementation of complex interventions. They concluded that the compatibility of a new intervention with the existing work routine was the most prevalent contextual factor in the implementation of new interventions. They stated “Some interventions were also perceived to be incongruent with habitual care routines and others were not deemed significantly different from existing practice to deserve a behavioral change” [39]. The guideline with recommendations enables GR teams to make a tailored plan for improvement, and only change their working method where it doesn’t align with the recommendations [39]. The implementation of our recommendations and advices can be supported by the development of an interdisciplinary training in which the GR teams undergo a critical self-reflection on the way their practice aligns to the recommendations, and in which they are trained in the goal setting conversation. Reflection on barriers and facilitators for the application of patient-centred goal setting should be a third component of the training. The review of Crawford et al. provides an extensive overview of this topic. [40]

Rather than choosing the best goal setting instrument, the guideline is built to solve the problems that hinder setting and achieving of proper goals. Most research only focusses on establishing goals together with the patients, and not on the role of the goals during rehabilitation [14, 17, 30, 35, 36]. Besides the study of Smit et al., we found a study in stroke rehabilitation about integration of goal setting all through the rehabilitation process, and the integration turned out to be poor [37]. Another study on this topic, is a pilot study that did not report results yet. [41] Our guideline strives for a central role for goals throughout the rehabilitation program, as this gives the patient control and ownership over his own rehabilitation, which is an important motivator according to professionals [35].

This study's PAR approach, developing an evidence-based guideline through collaboration between research and practice, is one of its strongest points. The collaboration between researchers and professionals within the GR committee of the UNO Amsterdam is an important facilitator for quality improvement by the development of evidence-based products for GR with a high feasibility. A second strength of this study is the choice for development of recommendations instead of a totally new method, thereby increasing the chance of successful implementation. A final strength of this study is the interdisciplinary focus on goals throughout the rehabilitation, by regularly and explicitly evaluating them with the patient and their relatives, and by explaining how each discipline contributes to the achievement of the goals.

A limitation of the study is the fact that the feasibility and the effect of the practical advices in the second cycle of PAR, are based on the analysis of a limited number of goal setting conversations.

Another weakness of this study is the fact that no patients were involved as co-researchers. The patient perspective was derived from scientific studies that reported on this perspective in the goal setting process. Besides, the participation of patients in the development of a goal setting intervention in adult and child rehabilitation resulted in similar topics for improvement as we found in our study [41]. The involvement of patients in the PAR would have further refined the recommendations.

Although the recommendations were developed in close contact with GR professionals and tested in daily practice, the effect on the quality of care has not been established yet. Future research should be conducted in co-creation with patients and their relatives. For example, relevant outcomes should be established in this co-creation process. In our opinion, studies should primarily focus on the effect on patient involvement in goal setting and patient ownership of the rehabilitation process. [42] Patient-related outcomes, such as improvements in physical functioning and quality of life, would be important secondary outcomes.

In conclusion, the practical guideline for goal setting in GR that was developed in this study, consists of eight recommendations and a further practical elaboration of three of them, concerning conversational skills that are specific for goal setting conversations. Multidisciplinary teams of GR professionals can use these recommendations for a tailored improvement of their patient centred goal setting practice. This can be supported by the development of an interdisciplinary training in which the GR teams undergo a critical self-reflection on the way their practice aligns to the recommendations, and in which they are trained in the goal setting conversation. The effect on quality of care should be subject to further investigation. Both the training and the research should be developed and conducted in co-creation with patients and their relatives.

### Supplementary Information

Below is the link to the electronic supplementary material.

Supplementary file1 (PDF 416 KB)

## Data Availability

The authors confirm that the evidence from literature is available within the article. For the video recordings, the participants of this study did not give written consent for their data to be shared publicly, so due to the sensitive nature of the research these data is not available.
