# Supplementary material for: Development of a practical guideline for person centred goal setting in geriatric rehabilitation: a participatory action research
Source: Eur Geriatr Med. 2023 Jul 17;14(5):1011–9. doi: 10.1007/s41999-023-00830-w (PMC10587279; doi:10.1007/s41999-023-00830-w)
Supplement: Supplementary file 1 — Supplementary file1 (PDF 416 KB) [file 41999_2023_830_MOESM1_ESM.pdf]

Online Resource A. Extensive description of the PAR-phases.

Phase	General	Cycle 1	Cycle 2
1	<u>Problem analysis</u><br>Members of the research team analysed the goal setting process combining both practical experience and literature research, and formulated the problem(s) that had to be solved.	No addition to general text.	Based on de outcome of Cycle 1, the research team decided to further elaborate the first three recommendations.
2	<u>Literature review</u><br>Members of the research team searched scientific literature to solve the problem(s) found in phase 1. The members stopped searching for new publications if they judged the new insights appropriate for solving the problems. The outcome of this phase were insights and ideas that can be used to solve the problems of phase 1	The literature search was meant to gain insights in the process of goal setting and to find new ideas to solve the problem(s) found in phase 1.	The research team gathered video recordings of goal setting conversation and searched for scientific literature on the following topics:<br>- Matching the patient's language;<br>- Shared decision making, and discussing the patient's role;<br>- Interaction between patients and professionals in goal setting conversations.
3	<u>Development</u><br>Members of the research team discussed the scientific literature in order to develop and subsequently adapt the guideline for goal setting in GR.	The research team discussed the scientific literature and developed recommendations for goal setting.	The research team discussed the literature and the video recordings and formulated practical advices for goal setting conversation skills.
4	<u>Practical experience</u><br>GR professionals (members of the research team and their colleagues in the GR organization) tested the guideline in their daily practice and provided feedback. The outcome of this phase was information on the feasibility and helpfulness of (a specific part of) the guideline to perform client centred goal setting, in terms of video recordings and /or the verbal or written feedback by the GR professionals.	GR professionals (members of the research team and their colleagues in the GR organization) tested the recommendations with the patients that were admitted to their GR ward. They received a brief instruction and recommendations for goal setting on paper. They gave verbal feedback to the research team.	GR professionals (members of the research team and their colleagues in the GR organization) tested the practical advices in a goal setting conversation with the patients that were admitted to their GR ward. They received a brief instruction and they received the practical advices presented in Box 2 on paper (without the comments in the right column).<br>Feedback was gathered by video recordings of the goal setting conversation and a short questionnaire on feasibility and helpfulness of the guideline for the GR professionals to perform client centred goal setting.
5	<u>Feedback and evaluation</u><br>Members of the research team reviewed the feedback and judged if the guideline was ready for dissemination and implementation in GR without further adjustments. This judgement was based on the feedback of the professionals (that tested the guideline).<br>If the outcome of phase 5 was that further adjustments to the guideline were necessary, a new cycle was started. Otherwise, the cyclical process was terminated.	The verbal feedback was discussed.	The video recordings of the patient interviews were assessed using a topic list checking if 1) the GR professionals applied the practical advices of Box 2 and 2) if they succeeded to comply to the first three recommendations of box 1.<br>The outcomes of the questionnaires for professionals were summarized.<br>All outcomes were discussed.

Title: Development of a practical guideline for person centred goal setting in geriatric rehabilitation – a participatory action research. **Journal**: European Geriatric Medicine.
Authors: Elizabeth M. Wattel, Aafke J. de Groot, Sacha Deetman-van der Breggen, Robin Bonthuis, Niels Jongejan, Marina M.R. Tol-Schilder, Johannes C. van der Wouden, Robbert Gobbens. **Corresponding author**: Elizabeth M. Wattel, Amsterdam UMC, location Vrije Universiteit Amsterdam, department of medicine for older people, Amsterdam, Netherlands. Amsterdam Public Health, Aging & Later Life, Amsterdam, Netherlands. l.wattel@amsterdamumc.nl.
